# A novel approach for effective superior vena cava isolation using the CARTO electroanatomical mapping system

**DOI:** 10.1002/joa3.12615

**Published:** 2021-08-13

**Authors:** Dai Inagaki, Seiji Fukamizu, Sayuri Tokioka, Takashi Kimura, Masao Takahashi, Takeshi Kitamura, Rintaro Hojo

**Affiliations:** ^1^ Department of Cardiology Tokyo Metropolitan Hiroo Hospital Tokyo Japan

**Keywords:** atrial fibrillation, CARTO system, extended early meets late, lower threshold, superior vena cava isolation

## Abstract

**Background:**

Previous studies have demonstrated that some patients have spontaneous right atrium (RA)‐superior vena cava (SVC) conduction block, which could be utilized to isolate the SVC effectively by using the Rhythmia mapping system (Boston Scientific). However, employing this approach for SVC isolation using the CARTO electroanatomical mapping system (Biosense Webster) has not yet been clarified. This study aimed to evaluate the safety and efficacy of SVC isolation using the extended early meets late (EEML) tool with the CARTO system.

**Methods:**

The patients who underwent SVC isolation using the CARTO system were enrolled in this study. The RA‐SVC conduction block was visualized with an EEML tool. We prospectively assessed the safety and efficacy of SVC isolation using this system.

**Results:**

We analyzed 54 patients, and all SVCs were successfully isolated with no complications. Altogether, 44 patients (81.5%) had spontaneous RA‐SVC conduction block, and the remaining 10 patients (18.5%) did not. The block group required fewer radiofrequency deliveries for the SVC isolation than the nonblock group (10.7 ± 5.0 vs 15.5 ± 4.8, *P* = .009). The size of the isolated area in the block group was larger than that in the nonblock group (15.2 ± 5.1 cm^2^ vs 12.4 ± 2.5 cm^2^, *P* = .017).

**Conclusions:**

Approximately 80% of the patients in this study developed a spontaneous RA‐SVC conduction block, which might contribute to shortening the time of ablation and avoiding complications.

## Graphical abstract

In our study, spontaneous RA‐SVC conduction block was observed in approximately 80% of the patients. The block group required fewer RF deliveries for SVC isolation than the non‐block group and the procedural time to SVC isolation in the block group was significantly less than in the non‐block group. The size of the isolated area in the block group was larger than that in the non‐block group.

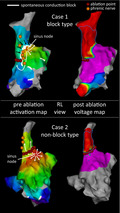

## 1 INTRODUCTION

Catheter ablation is a well‐established therapy for atrial fibrillation (AF).^1^, ^2^ Pulmonary veins (PV) are the most important AF sources; the superior vena cava (SVC) is one of the most important nonpulmonary vein origins of AF, and it can be identified in approximately 5% to 10% of patients who undergo AF ablation.^3^, ^4^ Previous studies have demonstrated that SVC isolation, in addition to PV isolation (PVI), could improve the clinical outcome of AF ablation.^5^, ^6^, ^7^ However, there is a risk of phrenic nerve and sinus node injuries because of radiofrequency (RF) application.^8^, ^9^, ^10^, ^11^ Hence, it is important to isolate SVCs safely and effectively without such complications.

Tanaka et al have demonstrated that some patients had spontaneous right atrium (RA)‐SVC conduction block, which could be utilized to isolate the SVC effectively by using an ultra‐high‐resolution mapping system (Rhythmia, Boston Scientific).^12^ They showed fewer RF deliveries for SVC isolation using the spontaneous conduction block than the conventional circumferential approach.

Recently, the CARTO 3 system (Biosense Webster) enabled automatic visualization of the conduction block as a white block line using the extended early meets late (EEML) tool. Using this function with the CARTO 3 system may achieve SVC isolation safely and effectively, similar to that with the Rhythmia system. This study aimed to evaluate the safety and efficacy of SVC isolation using the EEML tool with the CARTO system.

## 2 METHODS

### 2.1 Study protocol

A total of 63 consecutive patients with AF who underwent SVC isolation between September 2019 and September 2020 at the Tokyo Metropolitan Hiroo Hospital were enrolled in this prospective study. The study procedure complied with the principles of the Declaration of Helsinki and was approved by the Institutional Ethics Committee of the same hospital. A written informed consent was obtained from all the participants.

### 2.2 Catheter ablation

Antiarrhythmic drugs were discontinued for at least five half‐lives prior to the procedure. Oral anticoagulants were started at least 1 month before the procedure. The procedure was performed with continuous intravenous administration of propofol.

### 2.3 Pulmonary veins isolation

All patients underwent circumferential isolation of the four PVs at the antrum by either radiofrequency catheter ablation (RFCA) or cryoballoon ablation (CBA) during or prior to the SVC isolation session. Briefly, a decapolar catheter (Inquiry Luma‐Cath; St. Jude Medical) was inserted via the right subclavian vein into the coronary sinus. A transseptal puncture was performed using a radiofrequency‐powered needle under fluoroscopic and intracardiac echocardiographic (SOUNDSTAR; Biosense Webster or ViewFlex; St. Jude Medical) guidance. PV‐LA bipolar voltage mapping was performed using a multielectrode mapping catheter (Pentaray; Biosense Webster or Reflexion Spiral; St. Jude Medical) and a three‐dimensional anatomical mapping system (CARTO) before and after PVI. RFCA was performed using a ring catheter (LassoNav; Biosense Webster) and an irrigated‐tip ablation catheter (Thermocool SmartTouch; Biosense Webster). RF energy was delivered at 50 W, and the infusion rate was 17 mL/min via the irrigated‐tip ablation catheter and applied point‐by‐point to achieve a tag index of 500 for the anterior wall and 450 for the posterior wall. During PVI by CBA, a second‐generation 28‐mm cryoballoon (ARC‐Adv‐CB; Arctic Front Advance, Medtronic) with an inner lumen mapping catheter (Achieve, Medtronic) was inflated and advanced toward each PV orifice through a steerable 15‐F sheath (FlexCath Advance, Medtronic). Once optimal PV occlusion was achieved, as assessed by contrast injection, cryothermal energy was applied to each PV for 180 seconds. If –40°C or PVI was not achieved in 60 seconds, freezing was discontinued. If the PV potential remained after two CBA applications, touch‐up RF ablation via an irrigated‐tip ablation catheter (Thermocool SmartTouch or FlexAbility; St. Jude Medical or TactiCath; St. Jude Medical) was performed. A decapolar catheter was advanced into the superior vena cava, and the phrenic nerve was paced continuously during cryothermal energy application to the right superior and inferior PVs. The endpoint of PVI was the achievement of a bidirectional conduction block between the LA and PVs.

### 2.4 Mapping of the RA‐SVC

Electroanatomical RA‐SVC maps were created using the CARTO system during sinus rhythm to identify the location of the sinus node. Pentaray was used to collect the mapping data. The electrogram acceptance criteria were as follows: (a) position stability <2 mm, (b) local activation stability <3 ms, (c) maximum density. The length of the electrically activated SVC sleeve was measured from the RA‐SVC junction to the highest level of the SVC sleeve of which the voltage was >0.5 mV. The isolated SVC area was defined as the difference in the low‐voltage area before and after SVC isolation.

### 2.5 Setting the lower threshold in the activation map

A lower threshold (LT, %) was added to the CARTO 3 Version 6.0 system, which highlights areas of potential conduction block, called EEML. The value of the lower threshold can be set manually, although the optimal value is unknown.

We assessed the former cases to detect the ideal LT. We started ablation with low LT setting (5%), and if the SVC was not isolated by ablation to the gap of the visualized conduction block, the LT was increased by 5%, and the new gap was ablated each time. When the SVC was isolated, we decided that it was the real block line, and its LT was the optimal LT for its local activation time (LAT). The product of LAT and LT/100 indicates the conduction time at a given LT, and a white line is drawn where the conduction time between any two points is greater than that value. We assumed that the white line indicates the actual conduction block or slow conduction, if the LT was set to the optimal value. As a result of the retrospective evaluation of the 10 consecutive AF patients who underwent SVC isolation, the average value of the product of LAT and optimal LT/100 was 18.5 ± 3.2 ms. We assume that the conduction block exists where the local activation difference between adjacent points was more than this value. Hence, we set the first LT at 1800/LAT to optimize the visualization of spontaneous RA‐SVC conduction block and verified its validity.

### 2.6 SVC isolation

**FIGURE 1:**
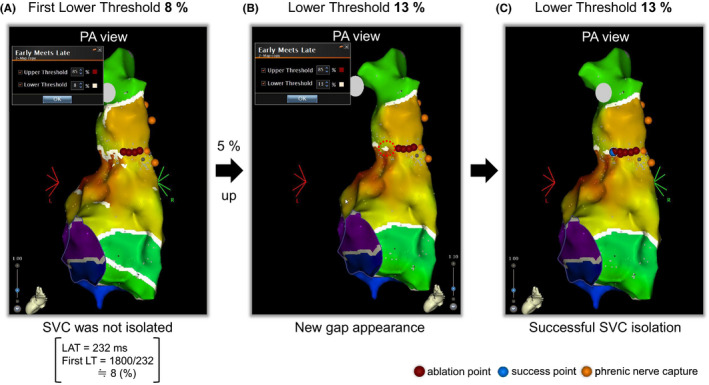
One of the cases of our ablation protocol (block type). A, We set the first lower threshold to the value of 1800/LAT to optimize visualization of the spontaneous RA‐SVC conduction block. In this case, the LAT is 232 ms, and we set the first lower threshold of 1800/232 ≒ 8 (%). RF application is delivered to the area where the white line does not appear but the SVC is not isolated. B, The lower threshold is 5% and increases to 13%, and a new gap appears. C, SVC isolation is successfully achieved through the additional ablation to the new gap. LAT, local activation time

**FIGURE 2:**
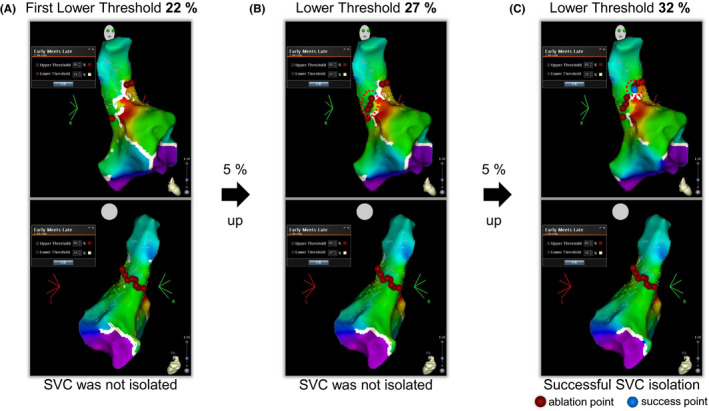
One of the cases of our ablation protocol (nonblock type). A, We set the first lower threshold of 22 (%). RF application is delivered to the area where the white line does not appear but the SVC is not isolated. B, The lower threshold is 5% and increases to 27%, and a new gap appears. RF application is delivered to the new gap but the SVC is not isolated. C, The lower threshold is 5% and increases to 32%, SVC isolation is successfully achieved through the additional ablation to the new gap

Briefly, a circular mapping catheter (LassoNav) was placed above the RA‐SVC junction. High‐output pacing was performed before ablation to avoid phrenic nerve injury. When the spontaneous conduction block was observed with the first LT set, RF energy was delivered point‐by‐point to the gap of the block line for 30 seconds at 30 W. If the SVC was not isolated by ablation to the gap of the visualized conduction block line, the lower threshold was increased by 5%, and the new gap was ablated each time (Figures 1 and 2). We classified the patients who needed the circumferential ablation eventually to achieve SVC isolation into the nonblock group. If a conduction block was not observed with the first LT, we also considered the case as a nonblock type. When necessary, RF applications were performed at 20 W on the phrenic nerve. The endpoint of SVC isolation was the achievement of a bidirectional conduction block between the RA and the SVC. After confirmation of the complete bidirectional block, continuous intravenous administration of isoproterenol (4 μg/min) was initiated, followed by a bolus injection of 40 mg of adenosine triphosphate (ATP) added to the isoproterenol infusion to exclude the presence of ATP‐provoked dormant conduction between the RA and SVC. Catheter ablation was performed to eliminate any reconnections and/or dormant conduction.

### 2.7 Statistical analysis

Data are presented as the mean ± standard deviation for continuous variables. Continuous variables were compared using the Student's *t* test for normally distributed variables. For categorical data, the chi‐squared test and Fisher's exact test were applied, as appropriate. All reported *P*‐values are two‐sided, with a *P*‐value <.05 was considered statistically significant. All statistical analyses were performed using the Statistical Package for Social Studies version 24 software (IBM Corp).

## 3 RESULTS

### 3.1 Clinical characteristics of the study patients

Of the 63 patients enrolled in this study, nine patients were excluded because of prior SVC isolation, ectopic atrial rhythm, or ablation during AF. Of the remaining 54 patients who were ultimately included and had paroxysmal AF (n = 26) and persistent AF (n = 28), 44 patients had a spontaneous RA‐SVC conduction block. Table 1 shows the baseline clinical characteristics of all patients, as well as those of the block and nonblock groups. The CHA_2_DS_2_‐VASc score was higher in the block group than in the nonblock group. The baseline characteristics of the patients included in this study were similar to those of the patients excluded from the study.

**TABLE 1:** Baseline clinical characteristics of all the patients, as well as in both the nonblock and block groups

	Total (n = 54)	block group (n = 44)	Nonblock group (n = 10)	*P*
Age, y	65.4 ± 10.7	66.5 ± 10.6	60.4 ± 10.1	.102
Female, n (%)	11 (20.4)	9 (20.5)	2 (20.0)	.974
Persistent AF, n (%)	28 (51.9)	24 (54.5)	4 (40.0)	.495
CHA_2_DS_2_‐VASc	2.1 ± 1.4	2.2 ± 1.5	1.5 ± 0.7	.036
Left atrium diameter, mm	37.2 ± 6.2	37.7 ± 5.7	34.7 ± 8.1	.162
Left atrium volume (TTE), mL	53.4 ± 20.1	55.0 ± 20.4	45.0 ± 17.5	.201
Left atrium volume (CT), mL	107.8 ± 25.1	107.2 ± 25.9	110.4 ± 22.4	.768
Ejection fraction, %	64.6 ± 6.7	64.9 ± 6.9	63.2 ± 6.2	.466

Note

Data are represent the mean ± SD.

Abbreviations: AF, atrial fibrillation; CT, computed tomography; TTE, transthoracic echocardiography.

### 3.2 RA‐SVC mapping

The mean number of mapping points was 620.0 ± 274.4. The earliest activation sites were located at the anterior (n = 16), anterolateral (n = 18), lateral (n = 16), and posterolateral walls (n = 4) of the patients with high RA. Of the patients, 44 had RA‐SVC spontaneous conduction blocks, and 10 patients did not (Appendices S1 and S2). Tanaka et al have defined the types of conduction block lines as follows: Type I: an oblique straight line of conduction block running from the lower lateral wall to the higher anterior wall; Type J: the conduction block line ran from the lower posterolateral wall to the higher midseptal wall in an arc with the highest portion at the anterior wall; Type U: block line that extended from the lower posterior to lower posteroseptal via the higher anterior wall around the RA‐SVC junction. Here, the types of conduction block lines observed in the patients were as follows: type I in 10 patients; type J in 15 patients; type U in 14 patients; and others in five patients (block line at the lateral to posterior wall in two patients and septal to posterior in two patients and block line drawn vertically at posterior wall in one patient) (Appendix S3). The RA‐SVC conduction block was higher in the anterior portion than in the posterior portion. There was no significant difference in the SVC sleeve length between the two groups (27.4 ± 7.8 mm vs 26.1 ± 9.4 mm, *P* = .651) (Table 2). The phrenic nerve was distributed only above the sinus node in 32 patients and ran from the SVC to below the sinus node in 22 patients.

**TABLE 2:** Procedural characteristics of the patients in both the nonblock and block groups

	Block group (n = 44)	Nonblock group (n = 10)	*P*
SVC sleeve length, mm	27.4 ± 7.8	26.1 ± 9.4	.651
Local activation time, s	112.1 ± 30.4	99.7 ± 13.1	.214
Lower threshold, %	17.1 ± 3.5	18.2 ± 2.3	.357
RA‐SVC mapping time, s	489.1 ± 165.2	610.0 ± 331.7	.097
Procedural time to SVC isolation, s	317.2 ± 155.7	465.0 ± 143.0	.008
Number of RF deliveries	10.7 ± 5.0	15.5 ± 4.8	.009
Isolated area, cm^2^	15.2 ± 5.1	12.4 ± 2.5	.017
Acute reconduction, n (%)	8 (18.2)	2 (20.0)	.894
Sinus node injury, n (%)	0 (0.0)	0 (0.0)	1
Phrenic nerve injury, n (%)	0 (0.0)	0 (0.0)	1

Note

Data represent the mean ± SD.

Abbreviations: RF, radiofrequency; SVC, superior vena cava.

### 3.3 SVC isolation

**FIGURE 3:**
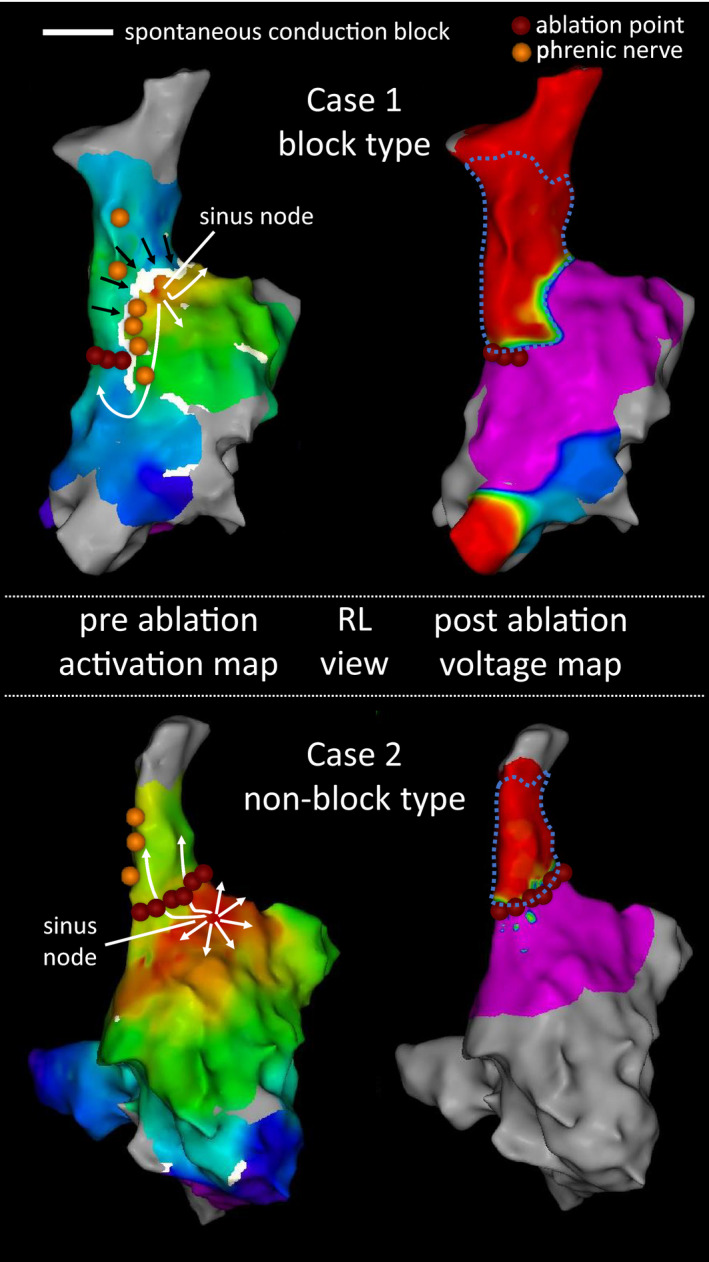
The representative cases of the block type and nonblock type. The block group requires fewer RF deliveries for SVC isolation than the nonblock group. RF application on the phrenic nerve is not required to achieve SVC isolation in the block group. Moreover, the size of the isolated area in the block group is larger than that in the nonblock group

**FIGURE 4:**
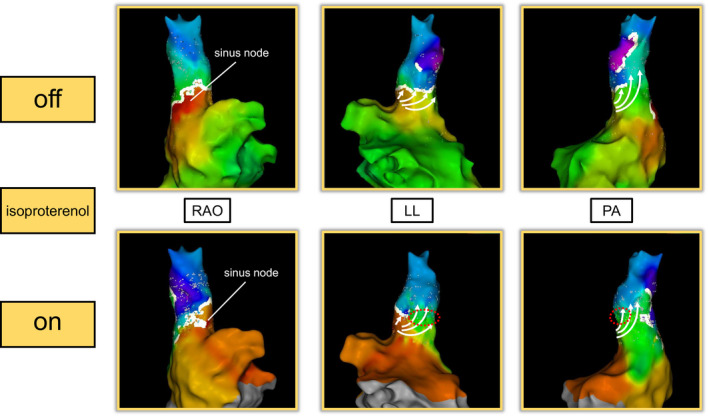
The representative case of RA‐SVC reconduction with isoproterenol. This is the case of the RA‐SVC reconduction with isoproterenol. Upper panels (without isoproterenol): Spontaneous conduction block (type U) appears, and RF application is delivered to the gap of the white line. Lower panels (with isoproterenol): RA‐SVC reconduction occurs with isoproterenol, and the former spontaneous conduction block disappears at the septal to posterior wall. SVC is successfully isolated by an additional RF application to the new gap

The block group required fewer RF deliveries for SVC isolation than the nonblock group (10.7 ± 5.0 vs 15.5 ± 4.8, *P* = .009) and the procedural time to SVC isolation in the block group was significantly less than in the nonblock group (317.2 ± 155.7 seconds vs 465.0 ± 143.0 seconds, *P* = .008). The size of the isolated area in the block group was larger than that in the nonblock group (15.2 ± 5.1 cm^2^ vs 12.4 ± 2.5 cm^2^, *P* = .017). Figure 3 shows examples of the block and nonblock groups. SVC isolation was successfully achieved with the first LT in 28 patients (51.9%). Of these patients, 20 SVCs (71.4%) were isolated only when all gaps were filled (four patients had acute reconduction). Acute reconduction with continuous intravenous administration of isoproterenol infusion was observed in 10 patients (eight in the block group and two in the nonblock group). Of these 10 patients, dormant conduction between the RA and SVC with a bolus injection ATP was observed in two patients. In one patient in the block group and in two patients in the nonblock group, acute reconduction with isoproterenol was observed at the posterior wall where the RF application had already been delivered. In seven patients in the block group, we successfully reisolated the SVC in all cases with ablation to the area of the disappeared spontaneous conduction block line (three at the lateral wall, three above the sinus node, one at the septal‐posterior wall; Figure 4). In the block group, no patient required RF application on the phrenic nerve to achieve SVC isolation. In the nonblock group, RF application was circumferentially delivered in five patients whose phrenic nerve was distributed only above the sinus node. In the remaining five patients whose phrenic nerve ran from SVC to below the sinus node, SVC was successfully isolated with circumferential RF application except for phrenic nerve site in four patients and RF application on the phrenic nerve was only needed in one patient. Neither sinus node injury nor phrenic nerve injury was observed.

## 4 DISCUSSION

The major findings of this study were as follows: (a) SVC isolation was effectively and safely achieved using the EEML tool of the CARTO system; (b) spontaneous RA‐SVC conduction block was observed in approximately 80% of the patients without isoproterenol; (c) SVC isolation was successfully achieved with the first LTs (1800/LAT) in approximately half of the patients; and (d) acute reconduction with continuous intravenous administration of isoproterenol infusion was observed in some patients in the block group, and most of them had RA‐SVC reconduction at the site of spontaneous RA‐SVC block without isoproterenol.

### 4.1 RA‐SVC junction

Here, the RA‐SVC conduction block was higher in the anterior portion than in the posterior portion, and atrial activation from the sinus node was conducted in a superior and clockwise direction as described in previous papers.^12^, ^13^, ^14^

Spontaneous RA‐SVC conduction block was observed in approximately 80% of the patients without isoproterenol, and we experienced reconduction of the RA‐SVC conduction block with isoproterenol infusion in eight patients in the block group. Previous report has demonstrated a case of isoproterenol‐dependent acute reconnection following SVC isolation using a spontaneous conduction block.^15^ They speculated that the reconduction with isoproterenol was because of pacemaker shift or disappearance of spontaneous functional block line, resulting in the difference in the percentage of the spontaneous conduction block here compared to that in previous reports.^12^ Another report have stated that with increasing heart rate, the site of the earliest endocardial depolarization remained stationary until a sudden shift in the cranial direction.^16^ We successfully reisolated the SVCs in seven of eight cases with ablation to the area where the original white line was. Based on these findings, it is important to reevaluate the conduction block under isoproterenol infusion. If we had used isoproterenol from the beginning of procedure, we might have been able to reduce acute reconduction between the RA and SVC. However, we did not use isoproterenol from the ablation initiation because of concerns on the unawareness of sinus node dysfunction. Moreover, the size of the isolated area can be larger without isoproterenol. It will take time, but it may be better if we can create electroanatomical RA‐SVC maps both under no medication and under isoproterenol infusion.

### 4.2 SVC isolation protocol

A previous study has demonstrated that SVC isolation was performed successfully and safely using the EEML of the CARTO system, although their ablation strategy was circumferential ablation, and phrenic nerve injury was observed in 4.5% of the patients in their study.^17^ Ivana et al have mentioned that the average thickness of the SVC sleeve of approximately 1 mm explains the relatively high risk of damage to the phrenic nerve during circumferential disconnection, so it might be advisable to restrict the application of RF energy only to sites of preferential conduction instead of anatomically guided circumferential disconnection.^18^ We did not ablate the spontaneous block line, and the block group required fewer radiofrequency deliveries for SVC isolation than the nonblock group, and the procedural time to SVC isolation in the block group was significantly less than that in the nonblock group. Moreover, most of the spontaneous conduction blocks appeared around the sinus node and phrenic nerve. Neither sinus node injury nor phrenic nerve injury was observed in this study. Goya et al have reported that the number of breakthroughs from the RA to the SVC was 1.4 ± 0.5, and RF delivery to the breakthrough points can isolate the SVC without unnecessary applications.^19^ Another study that assessed 620 formalin‐fixed hearts have reported that myocardial extensions into the SVC are common and less likely to be circumferential, suggesting that ablation for SVC isolation might not necessarily be circumferential.^20^ We agree that it is preferable to deliver a minimum RF application for SVC isolation from our experience. We experienced a relatively large number of reconductions with isoproterenol, most of which were found in the spontaneous block line. However, to avoid unnecessary application is one of the most important factors, and the rate of reconduction should not be a concern provided that we fully reassess the reconduction with isoproterenol and add the application properly if needed.

Previous study reported that dormant conduction between the RA and SVC can be exposed by ATP administration and we experienced that in two patients.^21^ A multicentre randomized trial described that adenosine testing to identify and target dormant pulmonary vein conduction during catheter ablation of AF is a highly effective strategy to improve arrhythmia‐free survival in patients with PAF.^22^ Considering the similarities between SVC and PV, it seems that dormant conduction between the RA and SVC should disappear and we successfully eliminate it.

### 4.3 Setting of the lower threshold

The ultra‐high‐resolution mapping system (Rhythmia) allowed the identification of the detailed pattern of SVC activation, which was useful for the safe and efficient SVC isolation during an AF ablation procedure.^12^ Compared with the Rhythmia system, the CARTO 3 system enables automatic visualization of the conduction block as a white block line by setting the LT manually. The value of the LT can be set manually, although the optimal value to visualize the real conduction block is unknown. Based on the former cases, we set the first LT to the value of 1800/LAT to optimize the visualization of spontaneous RA‐SVC conduction block and verified its validity. The threshold of conduction block or slow conduction was shorter than that of previous report (18.5 ms vs 24 ms).^17^ One of the reasons of this difference was that the conduction delay was objectively determined by reviewing past cases in this study, while it was determined subjectively in the previous study. In addition, we created the electroanatomical maps without isoproterenol, whereas in previous report, mapping was performed under isoproterenol administration, which may have contributed to conduction delay because of increased heart rate.

SVC isolation was successfully achieved with the first LT in approximately half of the patients. Of these patients, approximately 70% of the SVCs were isolated when all gaps were filled. This suggests that the visualized conduction block lines using this setting LT (1800/LAT) were real block lines in these patients. Although approximately half of the patients required an increase in LT, 1800/LAT may be a good value as the first LT to minimize wasteful ablation.

### 4.4 Study limitations

This study had limitations. First, the number of patients included in this study was relatively small and there was no control group. Further randomized studies with a larger number of patients are required. Second, as we only assessed the acute phase, the long‐term outcome was unclear. Third, SVC isolation was performed empirically in most patients who were included in this study; therefore, the clinical implications are unknown.

## 5 CONCLUSIONS

Approximately 80% of the patients had spontaneous RA‐SVC conduction block, which might contribute to shortening the time of ablation and avoiding complications.

## CONFLICT OF INTEREST

The authors have no financial conflicts of interest directly relevant to this study.

## Supporting information

Supplementary Material

Supplementary Material

Supplementary Material
