# Supplementary figures and images for: A novel approach for effective superior vena cava isolation using the CARTO electroanatomical mapping system
Source: J Arrhythm. 2021 Aug 13;37(5):1295–302. doi: 10.1002/joa3.12615 (PMC8485816; doi:10.1002/joa3.12615)

(A) Type I

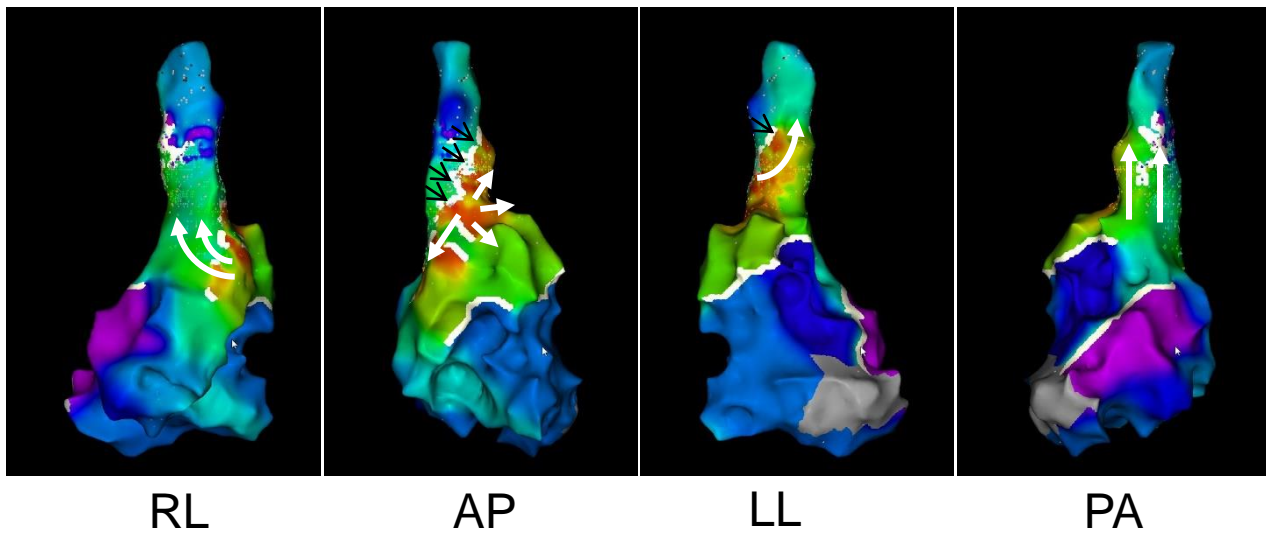

(C) Type U

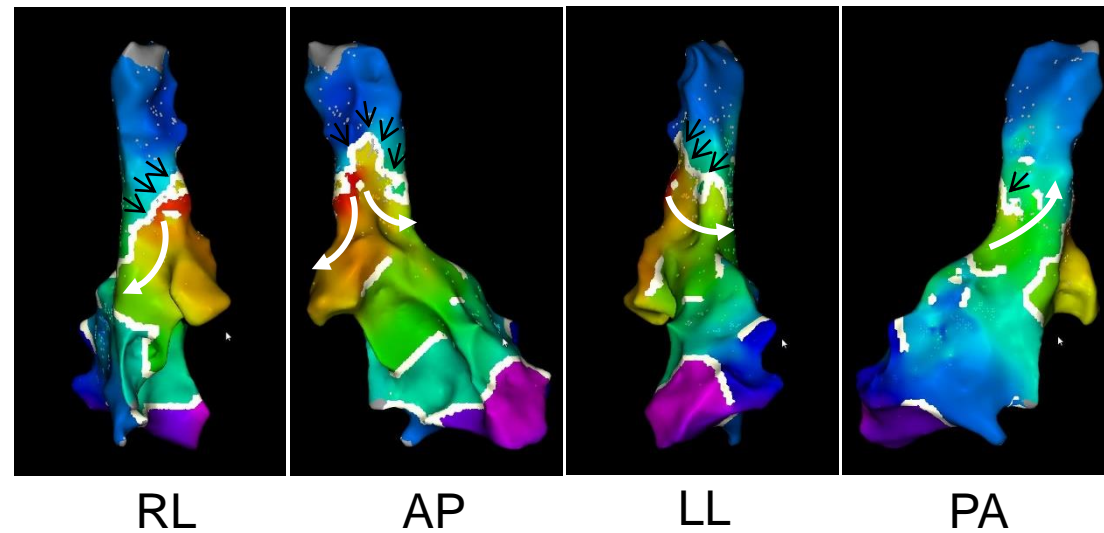

(B) Type J

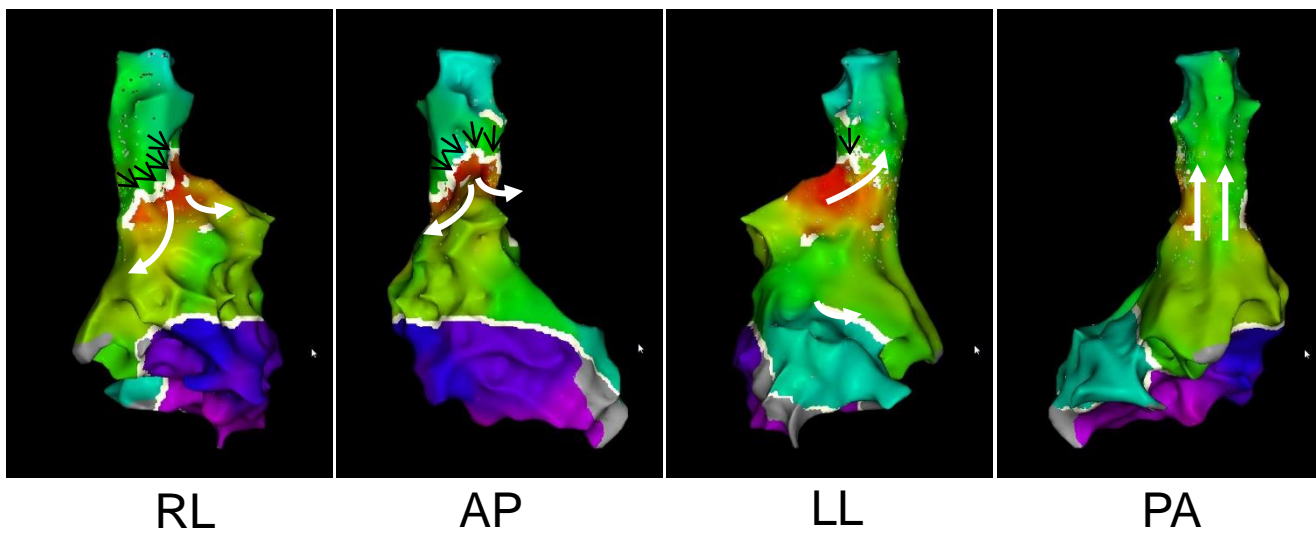

(D) Other

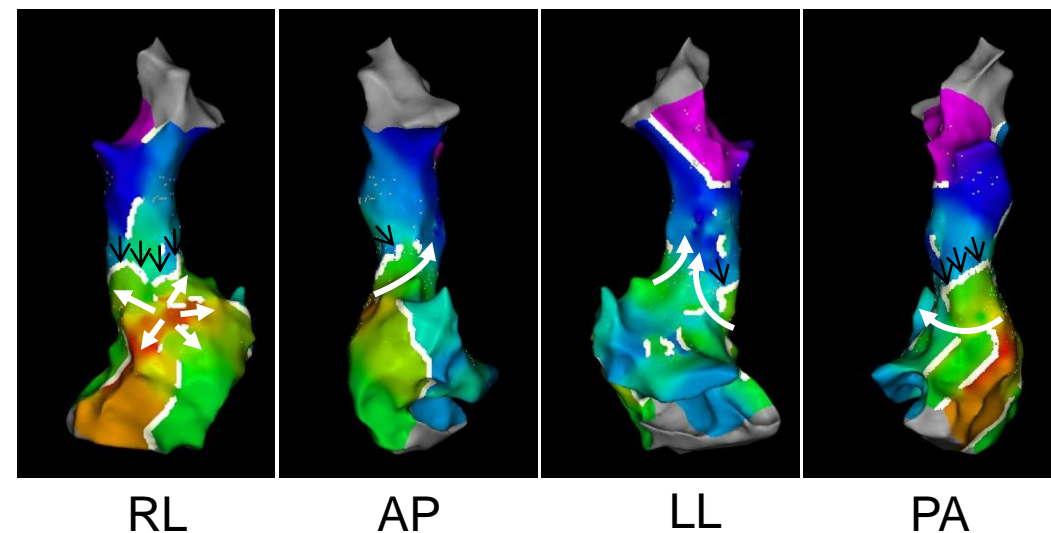

Supplement: Supplementary file 3 — Supplementary Material [file JOA3-37-1295-s002.pdf]
